# A Retrospective Study of Dexamethasone, Remdesivir, and Baricitinib in Severe COVID-19

**DOI:** 10.1155/2022/9209618

**Published:** 2022-07-06

**Authors:** Dallis Q. Ngo, Kewan Hamid, Haris Rana, Maria Cardinale, Douglas Frenia, Nabil Ghani, Henry Redel

**Affiliations:** ^1^Division of Pulmonary, Critical Care and Sleep Medicine, Saint Peter's University Hospital, New Brunswick, NJ, USA; ^2^Division of Pulmonary and Critical Care, University of South Alabama, Mobile, AL, USA; ^3^Division of Pharmacy, Ernest Mario School of Pharmacy, Rutgers The State University of New Jersey, Piscataway, NJ, USA; ^4^Division of Internal Medicine, Saint Peter's University Hospital, New Brunswick, NJ, USA; ^5^Division of Infectious Diseases, Saint Peter's University Hospital, New Brunswick, NJ, USA

## Abstract

**Purpose:**

RECOVERY, ACTT-1, and ACTT-2 trials have demonstrated that utilization of dexamethasone, remdesivir, or a combination of remdesivir with baricitinib leads to mortality benefit and faster time to recovery, respectively. However, no studies have investigated the benefit of triple therapy of dexamethasone, remdesivir, and baricitinib. We investigate the benefits of triple therapy compared to dual therapy of dexamethasone with remdesivir in patients with severe COVID-19 on HFNC.

**Materials and Methods:**

A retrospective data analysis was performed on patients with severe COVID-19 requiring HFNC and evaluated for hospital discharge status, requirement of mechanical ventilation, length of stay, and days on HFNC.

**Results:**

Among 191 patients with severe COVID-19, 81 patients received dexamethasone, remdesivir, and baricitinib. Patients receiving triple therapy had a significant survival benefit (HR 0.52; *P*=0.042). Treatment with triple therapy vs. dual therapy also trended towards less requirement of mechanical ventilation (OR 0.66; *P*=0.26). There was no significant change in length of stay (mean 13.74 vs. 13.31; *P*=0.74) or days on HFNC (mean 8.95 vs. 7.28 days, *P*=0.16).

**Conclusions:**

The use of dexamethasone, remdesivir, and baricitinib in patients with severe COVID-19 requiring HFNC was associated with a significant survival benefit in comparison to dual therapy of dexamethasone with remdesivir.

## 1. Introduction

In December 2019, a novel coronavirus identified as severe acute respiratory syndrome coronavirus 2 (SARS-CoV-2) was identified as the cause of a deadly global pandemic, inflicting a respiratory illness termed COVID-19 [1]. Although the outbreak was likely to have originated from zoonotic transmission occurring at markets where live wild animals are traded, it became clear that person-to-person transmission was also occurring [2, 3]. At the start of 2021, the global COVID-19 case count exceeded 89.2 million individuals with 1.92 million deaths. These numbers continued to rise, and by the time of this study's completion, the death toll had risen to 4.36 million individuals [4]. Although the majority of COVID-19 cases across the world have either been asymptomatic or associated with mild disease, a substantial percentage of cases progress and lead to hypoxic respiratory failure requiring hospitalization for oxygen therapy [2]. This has been classified as a “severe disease” and defined as individuals with COVID-19 requiring mechanical ventilation or supplemental oxygen, having a SpO_2_ ≤94% breathing ambient air, or tachypnea ≥24 breaths per minute [1]. Severe COVID-19 infection has been associated with patchy peripheral opacities on radiographs, inflammatory alveolar infiltrates, and microvascular thrombosis, which is thought to be related to marked elevation of inflammatory markers.

During the COVID-19 pandemic's infancy, studies of infected patients reported findings of elevated levels of proinflammatory cytokines (e.g., IL-1, IL-2, IL-6, IL-12, IL-18, and TNF-*α*) that eventually cause uncontrolled systemic inflammation and multiorgan damage [5]. This finding is now commonly referred to as cytokine storm and led to comparison to another syndrome in critical illness associated with elevated cytokines termed cytokine release syndrome (CRS) [6]. For this reason, tocilizumab, a humanized monoclonal antibody against IL-6 receptors and commonly used for the treatment of CRS, was one of the first agents to be used for treatment of severe COVID-19. The clinical benefit of tocilizumab varies, with some studies suggesting mortality benefits in those with the most severe disease [6, 7]. Multiple studies have since emerged, investigating agents that could curtail the inflammatory response to COVID-19.

The RECOVERY trial demonstrated that treatment with oral or intravenous dexamethasone at a dose of 6 mg once daily for up to 10 days was superior to placebo, resulting in lower 28-day mortality among those who required invasive mechanical ventilation or supplemental oxygen therapy [8]. Currently, the benefit of utilization of dexamethasone, or equivalent doses of other systemic corticosteroids to mitigate inflammatory organ injury, has led to a strong recommendation for its use from the WHO [9]. In October 2020, the ACTT-1 trial was published. The study tried to show the effect of remdesivir on time to recovery. Remdesivir is an inhibitor of the viral RNA-dependent, RNA polymerase which has shown inhibitory activity in prior studies against SARS-CoV-1 and MERS-CoV [1]. In the ACTT-1 trial, remdesivir was administered intravenously as a loading dose of 200 mg on day 1, followed by 100 mg daily on day 2 through 10 or until hospital discharge. In comparison to the placebo group, those treated with remdesivir had a shorter time to recovery with a median of 10 days vs. 15 days, especially in patients who required low-flow oxygen therapy [1]. The benefit of remdesivir in recovery time persisted despite adjustment for glucocorticoid use, suggesting additive benefit. Dual therapy of dexamethasone with remdesivir has since become the standard of care for treatment of severe COVID-19 pneumonia in the USA [10].

In December 2020, the ACTT-2 trial was published. The ACTT-2 investigators utilized a combination of remdesivir with baricitinib for treatment of adult patients hospitalized with COVID-19. Baricitinib is an oral medication that selectively inhibits Janus kinase (JAK) 1 and 2 that inhibit intracellular cytokine pathways (IL-2, IL-6, IL-10, IFN*γ*, and GM-CSF) elevated in severe COVID-19 infection as well as prevent cellular entry/infectivity of SARS-CoV-2 by impairing AP2-associated protein kinase 1 [11]. The combination of baricitinib with remdesivir was found to be superior to remdesivir alone in reducing time to recovery in patients with COVID-19 and, especially in those requiring high-flow oxygen or noninvasive ventilation.

The next step in advancing the field of medicine and care for the patient inflicted with COVID-19 would be to investigate the utility of a combination of dexamethasone, remdesivir, and baricitinib. We performed a retrospective prepost study to evaluate the efficacy of triple therapy of dexamethasone, remdesivir, and baricitinib in comparison to conventional dual therapy of dexamethasone with remdesivir in severe COVID-19 requiring high-flow oxygen.

## 2. Methods

The protocol was designed and written by the investigators and approved by the Saint Peter's University Hospital Institutional Review Board. The study is nonsponsored. The investigators at the participating study site gathered the data, while the first and second authors performed the statistical analysis. The authors equally wrote the entire manuscript and vouch for the accuracy, completeness of the data, and fidelity to the study protocol.

We performed a single-site, retrospective cohort study conducted at a community-based tertiary care hospital in New Brunswick, NJ, USA. Patients having symptoms suggestive of COVID-19 infection were screened at the emergency department for COVID-19 infection. Based on the severity of patient symptoms and clinical judgement of emergency medicine physicians, patients with severe disease were classified as needing ICU care or lower-level care. Patients with COVID-19 admitted between June 24, 2020, and June 1, 2021, were screened for the inclusion and exclusion criteria of the study. The full inclusion criteria included male or nonpregnant female with age ≥18 years old at the time of hospital admission, admitted to a hospital with symptoms suggestive of COVID-19, had a laboratory-confirmed SARS-COV-2 infection determined by PCR, illness of any duration with evidence of lower respiratory tract infection via radiographic infiltration by chest radiography and SpO_2_ ≤94%, and required high-flow oxygen evident by a score of 6 on the ordinal scale (hospitalized, on noninvasive ventilation, or high-flow oxygen devices). Patients were excluded from the study if they met any of the following criteria: ALT or AST >5 times the upper limit of normal, neutropenia (absolute neutrophil count <250 cells/*μ*L), pregnancy or breastfeeding, anticipated discharge from the hospital or transfer to another hospital which is not a study site within 72 hours, allergy to study medication, received TNF inhibitor within 2 weeks of screening, received convalescent plasma or intravenous immunoglobulin for COVID-19, or received mechanical ventilation within 24 hours of admission. Baseline characteristics of the treatment and control groups are listed in Table 1.

Patients in the control group were treated with dexamethasone 6 mg IV daily for 10 days and remdesivir 200 mg IV once followed by 100 mg IV daily for 5 days total. Those in the treatment group received baricitinib 4 mg oral daily for 14 days in addition to dexamethasone and remdesivir. Treatment could be discontinued early if the patients were discharged from the hospital due to recovery. Patients could receive tocilizumab as the standard of care for management of severe COVID-19 requiring high-flow oxygen at the discretion of the providing physician if they were perceived to be at a periintubation stage and high risk for requiring mechanical ventilation.

Using Saint Peter's University Hospital data registry, the investigators involved in this study gathered the clinical data, which were then analyzed using STATA© 15.0 (College Station, Texas, USA). The study was approved by the Institutional Review Board (IRB) of Saint Peter's University Hospital (IRB# 00004301). Exception from obtaining informed consent was granted by the IRB considering our study was retrospective in nature.

The study's primary outcome measure was the patient discharge status. A Cox proportional hazard regression model was used to compare the treatment group treated with dexamethasone, remdesivir, and baricitinib versus the control group treated with dexamethasone and remdesivir. In addition, the recovery status in patients requiring mechanical ventilation regardless of the treatment group was analyzed. The secondary outcome measures included hospital's length of stay and need for positive pressure ventilation between the two groups. Logistic regression analysis was conducted to compare the two groups in regards to age, gender, race, BMI, and need for mechanical ventilation. Safety outcomes between the two study arms were not compared during this study. Statistical significance is determined as a *P* value of less than or equal to 0.05.

## 3. Results

A total of 232 patients were screened for eligibility, and 191 patients were enrolled after meeting our eligibility criteria. One hundred ten patients were placed in the control group, while 81 patients were placed in the treatment group. There was no statistical difference in regards to distribution of BMI, age, gender, race, or medical comorbidities between the two arms of the experiment (Table 1).

**Figure 1:**
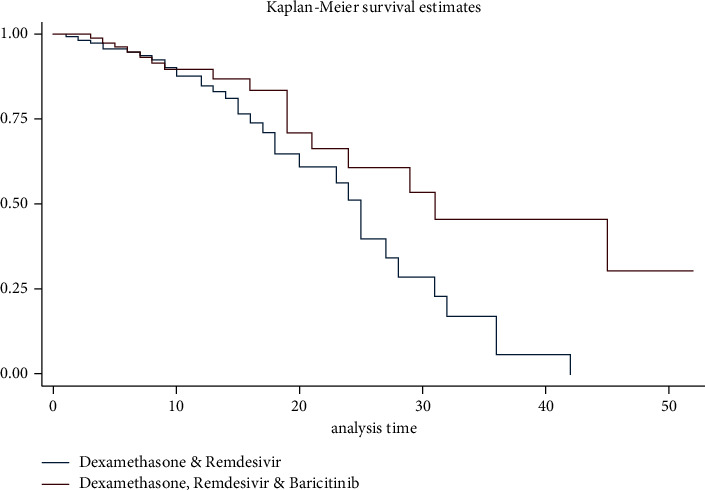
Kaplan–Meier survival estimates between control and experimental arms.

**Figure 2:**
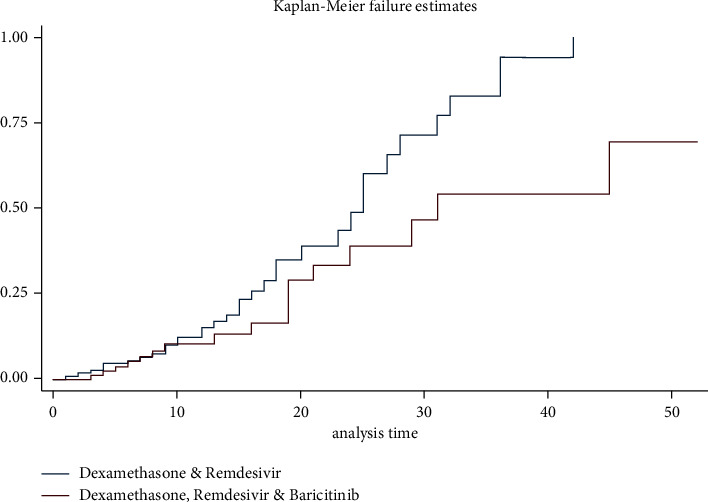
Kaplan–Meier failure estimates between control and experimental arms.

In regards to the primary outcome of the study, those that received a combination of dexamethasone, remdesivir, and baricitinib had a statistically significant higher survival rate than those in the control group (hazard ratio, 0.53; *P*=0.04; 95% CI, 0.29–0.97) (Table 2, Figures 1 & 2). The requirement for mechanical ventilation was associated with a statistically significant decrease in survival (hazard ratio, 2.32; *P*=0.005; 95% CI, 1.28–4.19) (Table 2). The secondary outcome showed those in the treatment group needed less mechanical ventilatory support when compared to the control group (14 vs. 28 patients); however, this difference in distribution was not statistically significant (odds ratio, 0.66; *P*=0.26; 95% CI, 0.31–1.36) (Table 3). Patients in the treatment group remained longer on high-flow oxygen than those in the control group; however, this difference was not statistically significant (mean 8.95 vs. 7.28 days; *P*=0.16) (Table 3). Inpatient length of stay was similar between the control and treatment groups (mean 13.31 vs. 13.74 days, *P*=0.74) (Table 3).

**Figure 3:**
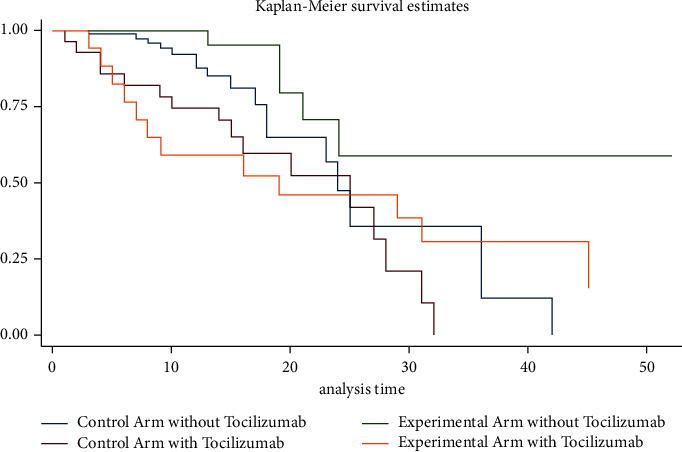
Kaplan–Meier survival estimates in study arms separated by tocilizumab usage.

Tocilizumab was administered to 17 patients (20.99%) in the experimental arm and 28 patients (25.45%) in the control arm; however, there was no statistical difference in distribution between the two arms of the study (odds ratio 0.93; *P*=0.86). Regardless of the study arms, patients who received tocilizumab spent more mean days on mechanical ventilation and high-flow oxygen than those who had not (5.80 vs. 1.31 days, *P* ≤ 0.001) (10.29 vs. 7.28, *P*=0.03). However, since patients who required high-flow oxygen and mechanical ventilation overlapped, regression analysis was conducted to evaluate these findings and showed that days on mechanical ventilation remained statistically significant (OR 1.11; *P* ≤ 0.001; 94% CI 1.05–1.18), while days on high-flow oxygen did not (OR 1.03; *P*=0.14; 95% CI 0.99–1.07). The overall median survival in days was 31 in the experimental arm versus 25 in the control arm. Compared with the control arm, there was a statistically significant increase in survival in the experimental arm when patients receiving tocilizumab were eliminated from both groups (HR 0.36; *P*=0.04; 95% CI 0.13–0.96) (Figure 3). No statistically significant difference in survival was observed in those who had received tocilizumab in addition to a standard treatment protocol in both arms of the study (HR 0.77, *P*=0.54, 95% CI 0.34–1.73).

## 4. Discussion

The results of this retrospective cohort study successfully demonstrate that patients with severe COVID-19 requiring high-flow oxygen derive a significant survival benefit when treated with a triple therapy combination of dexamethasone, remdesivir, and baricitinib, in comparison to a combination of dexamethasone and remdesivir. This is the first study focused on COVID-19 patients requiring high-flow oxygen to successfully demonstrate this finding. Amelioration of viral diseases that cause systemic inflammation relies on two strategies: reducing viral entry and replication in the target tissue and reducing inflammation to curtail organ damage [12, 13]. Glucocorticoid use in COVID-19 accomplishes the latter through broad suppression of proinflammatory cytokines. An important concept to not forget is that suppression of the immune system and inflammatory response via glucocorticoids also weakens the defense against viral infection, increasing a virus's ability to infect and replicate. This has been shown to be true and potentially fatal in sepsis secondary to influenza and bacterial infections [14, 15]. Despite this, glucocorticoids continue to confer a survival benefit in COVID-19 patients as a consequence of what is believed to be due to viral-induced glucocorticoid insensitivity. Studies have shown that similar to rhinovirus, SARS-CoV-2 infection activates transcription factors such as nuclear factor-*κ*B (NF*κ*B) and activator protein-1 (AP-1) to suppress glucocorticoid receptor activity, causing glucocorticoid insensitivity in virus-infected cells [13, 16]. Based on the prior success for use against SARS-CoV-1 and MERS-CoV viruses, remdesivir use achieves clinical benefit against SARS-CoV-2 through the former of the two strategies by inhibiting viral RNA-dependent RNA polymerase (RdRp), causing premature termination of viral RNA transcription [17–20]. Remdesivir, in mouse models, produced greater than two orders of magnitude reduction of the pulmonary viral load and mitigates disease progression [19, 21]. Human clinical trials showed a significant reduction in recovery time [1]. The benefits of baricitinib use arise from utilizing both the former and latter defensive strategies to ameliorate viral disease. Baricitinib, a JAK-1 and JAK-2 inhibitor, blocks the signaling pathway of cytokine release responsible for cytokine storm as well as prevention of SARS-CoV-2 cellular entry via impairment of AP2-associated protein kinase 1 [11, 22]. The use of baricitinib when combined with remdesivir in hospitalized COVID-19 adults requiring high-flow oxygen or noninvasive mechanical ventilation showed a significant improvement in time to recovery. We believe that the combination treatment of dexamethasone, remdesivir, and baricitinib was able to show additional improvement likely due to an additive effect by targeting multiple pathways of the disease process. Our findings remain consistent with the mortality benefit shown in a prior study of baricitinib coadministration with corticosteroids, remdesivir, and/or an IL-6 receptor blocker in COVID-19 patients predominantly requiring low-flow oxygen [23].

However, we did observe that the requirement of mechanical ventilation was associated with a statistically significant decrease in survival of all patients regardless of treatment, and there was no significant difference in the number of patients requiring mechanical ventilation between patients treated with triple therapy or dual therapy. There is, however, a trend towards a reduction in the requirement of mechanical ventilation for those treated with triple therapy (17.3% vs. 25.5%). This may be due to insufficient power of the study for the secondary outcome. However, the benefit of an 8.2% reduction in mechanical ventilation by coadministration of baricitinib in our study is consistent with the 5.2% reduction seen in the ACTT-2 trial [11].

The benefit from addition of baricitinib to conventional dual therapy of dexamethasone and remdesivir was not observed in regards to the mean days on high-flow oxygen. In comparison to prior studies, our findings regarding the median days on high-flow oxygen in the control group are consistent with those found in the dexamethasone remdesivir group presented by the ACTT-1 investigators (5 vs. 6 days) [1].

Multiple studies have defined recovery as the first day in which patients attained category 1, 2, or 3 on the 8-point ordinal scale [1, 11]. This can be related to our study when observing length of inpatient stay as none of our patients required additional hospital days for nonmedical care during the chart review. We were unable to demonstrate a significant difference in length of inpatient stay with treatment of triple therapy versus dual therapy, likely as a result of the study being underpowered to detect a significance difference. Our median length of stay of 11 days in the test group is consistent with the baricitinib remdesivir group presented by the ACTT-2 investigators [11].

Patients in our study received tocilizumab as it was the standard of care at the time of study date if the providing physician determined that the patient was at a periintubation stage with a high risk of further deteriorating and requiring mechanical ventilation. Both experimental and control arms had similar usage of tocilizumab. Those that received tocilizumab were sicker as reflected by a statistical longer mean duration on mechanical ventilation and reduced survival as seen on the Kaplan–Meier curve in Figure 3. Contrasting prior studies, no significant survival benefit was observed with tocilizumab usage; however, this may be due to a smaller sample size and lack of statistical power [24]. A prior meta-analysis studying cytokine levels of severe COVID-19 patients with cytokine storm revealed that the mean serum IL-6 concentration was 36.7 pg/mL, 100 times lower than patients with cytokine release syndrome, 27 times lower than patients with sepsis, and 12 times lower than patients with ARDS unrelated to COVID-19 [6]. The lack of benefit of tocilizumab in our study and mixed results in the current literature suggest that COVID-19 does not cause a severe cytokine response as CRS and therefore only benefits a small subset of patients [6, 7]. Cytokine levels were not measured in our study. The statistically significant survival benefit of combination of dexamethasone, remdesivir, and baricitinib usage was seen even after exclusion of patients who had received tocilizumab.

Our study has several limitations including the single-centered, retrospective nature leading to a small sample size. The presence of tocilizumab usage in our study population makes conclusions regarding the benefit of triple-drug therapy of dexamethasone, remdesivir, and baricitinib less clear-cut. However, the decision to include this population was determined based on the fact that tocilizumab usage was the standard of care at the time of the study for treatment of severe COVID-19 requiring high-flow oxygen. Exclusion of this population would lead to exclusion of a sicker cohort and would not reflect a general hospitalized population of severe COVID-19. Another limitation is that the patients were treated with each regimen at different times. The circulating SARS-CoV-2 strains were different at the time and may have had an impact on clinical outcomes when comparing different times. This has not been reported to date, and our study occurred prior to the emergence of the delta variant. In addition, the patients in the control group had a higher rate of chronic kidney disease, implying patients might have been sicker in this group. Our rationale to limit the study to a single site was to minimize variables such as different COVID-19 management practices, variability in availability of medications, ventilatory support devices, medical staff, and burden on the medical system. Future avenues of investigation could include randomized control trials comparing rates of secondary infection as triple therapy of dexamethasone, remdesivir, and baricitinib becomes more common.

## 5. Conclusion

The use of combination dexamethasone, remdesivir, and baricitinib in patients with severe COVID-19 requiring high-flow oxygen therapy was associated with a significant survival benefit in comparison to dual therapy of dexamethasone with remdesivir. Triple therapy is not associated with a significant difference in the need for mechanical ventilation in comparison to the control group.

## Figures and Tables

**Table 1:** Baseline demographic and clinical characteristics.

	Baricitinib + dexamethasone + remdesivir *n* = 81	Dexamethasone + remdesivir *n* = 110	Odds ratio	*P* value	95% CI
BMI			1.01	0.55	0.97–1.05
Mean	31.87 ± 0.78	30.36 ± 0.90			
Median	31.7	29.75			

Age			0.99	0.38	0.97–1.01
Mean	61.67 ± 1.76	64.68 ± 1.67			
Median	62	63			

Gender (%)			0.56	0.07	0.29–1.05
Male	50 (61.73)	82 (74.55)			
Female	31 (38.27)	28 (25.45)			

Race (%)			0.95	0.37	0.86–1.05
American Indian or Alaskan native	1 (1.23)	1 (0.91)			
Asian	3 (3.7)	1 (0.91)			
Asian Indian	7 (8.64)	5 (4.55)			
Black	14 (17.28)	23 (20.91)			
Filipino	2 (2.47)	3 (2.73)			
Samoan	0 (0)	2 (1.82)			
Other Pacific islander	2 (2.47)	1 (0.91)			
White	40 (49.38)	63 (57.27)			
Unknown	2 (2.47)	0 (0)			
Patient declined	10 (12.35)	11 (10)			

Ordinal score (%)					
4 = hospitalized, not requiring supplemental oxygen, requiring ongoing medical care (COVID-19 related or otherwise	3 (3.7)	10 (9.09)			
5 = hospitalized, requiring supplemental oxygen	25 (30.86)	48 (43.64)			
6 = hospitalized, on noninvasive ventilation or high-flow oxygen devices	53 (65.43)	52 (47.27)			

Pre-existing comorbidities (%)					
Asthma	6 (7.41)	6 (5.45)	1.52	0.5	0.45–5.10
Congestive heart failure	7 (8.64)	14 (12.73)	0.86	0.79	0.29–2.53
Chronic kidney disease	9 (11.11)	24 (21.81)	0.46	0.1	0.18–1.16
Chronic obstructive pulmonary disease	7 (8.64)	9 (8.18)	1.2	0.74	0.41–3.53
Diabetes	36 (44.44)	42 (38.18)	1.53	0.19	0.81–2.87
Hyperlipidemia	41 (50.61)	47 (42.73)	1.62	0.16	0.83–3.16
Hypertension	44 (54.32)	68 (61.82)	0.63	0.19	0.32–1.25
Hypothyroidism	11 (13.58)	11 (10)	1.62	0.31	0.64–4.11
Tocilizumab (%)	17 (20.99)	28 (25.45)	0.93	0.86	0.43–2.03

**Table 2:** Primary outcomes.

	Hazard ratio	*P* value	95% CI
Discharge status (dead vs. alive)	0.53	0.042	0.29–0.97
Requirement of mechanical ventilation on discharge status	2.32	0.005	1.28–4.19

**Table 3:** Secondary outcomes.

	Baricitinib + dexamethasone + remdesivir *n* = 81	Dexamethasone + remdesivir *n* = 110	Odds ratio	*P* value	95% CI
Requirement of mechanical ventilation			0.66	0.26	0.31–1.36
Not requiring mechanical ventilation (%)	14 (17.3)	28 (25.5)			
Mean (days)	10.9	10.7			
Median (days)	9.5	9.5			
Length of stay (days)			*t *− 0.39	0.74	
Mean (95% CI)	13.74 (11.44–16.04)	13.31 (11.89–14.73)			
Median	11	12			
High-flow oxygen (days)			*t *− 1.39	0.16	
Mean (95% CI)	8.95 (6.91–10.99)	7.28 (5.91–8.65)			
Median	7	5			

## Data Availability

Data access is restricted due to legal and ethical concerns, such as third-party rights and patient privacy.

## Additional Points

The novel coronavirus identified as SARS-CoV-2 in December 2019 has led to a deadly worldwide pandemic that has ravaged the health of the global population. By 2021, the COVID-19 case count exceeded 89.2 million individuals and 1.92 million deaths globally. Numerous landmark studies in the fight against COVID-19 have investigated the individual benefits of dexamethasone, remdesivir, and baricitinib for treatment of severe COVID-19. However, no studies have been conducted to investigate the benefits of triple therapy of all three drugs. We performed a retrospective study investigating the effects of triple therapy in patients with severe COVID-19 pneumonia who require high-flow oxygen.
